# Lipid Metabolic Reprogramming in Embryonal Neoplasms with MYCN Amplification

**DOI:** 10.3390/cancers15072144

**Published:** 2023-04-04

**Authors:** Jyotirmayee Talapatra, Mamatha M. Reddy

**Affiliations:** 1 The Operation Eyesight Universal Institute for Eye Cancer, L V Prasad Eye Institute, Bhubaneswar 751024, India; 2 School of Biotechnology, KIIT Deemed to Be University, Bhubaneswar 751024, India

**Keywords:** cancer, embryonal tumors, MYCN, lipid metabolism, therapeutic targeting

## Abstract

**Simple Summary:**

Cancer cells exhibit unrestrained cell growth and proliferation owing to tumor-specific characteristics such as altered metabolism. Tumor cells vary metabolic pathways to meet their needs for rapid cell growth. In this review, we focus particularly on lipid metabolism in embryonal tumors with deregulated MYCN function, specifically neuroblastoma, retinoblastoma, Wilms tumor, medulloblastoma, and rhabdomyosarcoma. We also discuss whether we can target lipid metabolism to restrict tumor cell growth and proliferation.

**Abstract:**

Tumor cells reprogram their metabolism, including glucose, glutamine, nucleotide, lipid, and amino acids to meet their enhanced energy demands, redox balance, and requirement of biosynthetic substrates for uncontrolled cell proliferation. Altered lipid metabolism in cancer provides lipids for rapid membrane biogenesis, generates the energy required for unrestricted cell proliferation, and some of the lipids act as signaling pathway mediators. In this review, we focus on the role of lipid metabolism in embryonal neoplasms with MYCN dysregulation. We specifically review lipid metabolic reactions in neuroblastoma, retinoblastoma, medulloblastoma, Wilms tumor, and rhabdomyosarcoma and the possibility of targeting lipid metabolism. Additionally, the regulation of lipid metabolism by the MYCN oncogene is discussed.

## 1. Introduction

The embryonal neoplasms with MYCN (myelocytomatosis-neuroblastoma derived) protooncogene amplification are predominantly malignant and show distinct characteristic features. MYCN is a member of the MYC transcription factor family. MYC family consists of three paralogs, c-MYC, MYCN, and L-MYC. The first human MYC gene, which encodes the oncoprotein c-MYC, was discovered to be the cellular homolog of the avian retroviral transforming gene, v-MYC (viral myelocytomatosis) [1,2,3]. MYCN was identified as a paralog of c-MYC in neuroblastoma cell lines and tumor tissues [4]. The L-MYC gene was initially discovered in small-cell lung cancer based on homology to both c-MYC and MYCN [5]. MYC proteins regulate the expression of approximately 15% of all human genes [6]. MYC proteins activate and/or repress transcription of several genes involved in normal cellular processes such as cell growth, cell cycle progression, proliferation, survival, pluripotency, self-renewal, angiogenesis, metabolism, and apoptosis [7,8,9].

Amplification of MYC family members is the most common event that contributes to the deregulation of MYC in human cancers. Gene duplication via genome doubling or tandem duplications could be likely mechanisms for copy number variations (CNVs) or oncogene amplification [10]. MYCN amplification was first identified in neuroblastoma (NB) cell lines and tumor specimens [4,11]. Both c-MYC and MYCN were found to be associated with NB, however, poor outcomes were observed with MYCN amplification [12,13]. Small cell lung cancer (SCLC) was the first cancer found to have amplification of all three members (c-MYC, L-MYC, and MYCN) of the MYC family of proto-oncogenes [5,14,15]. MYCN likewise was found to be deregulated in embryonal tumors [16].

## 2. MYCN Structure and Function

**Figure 1:**
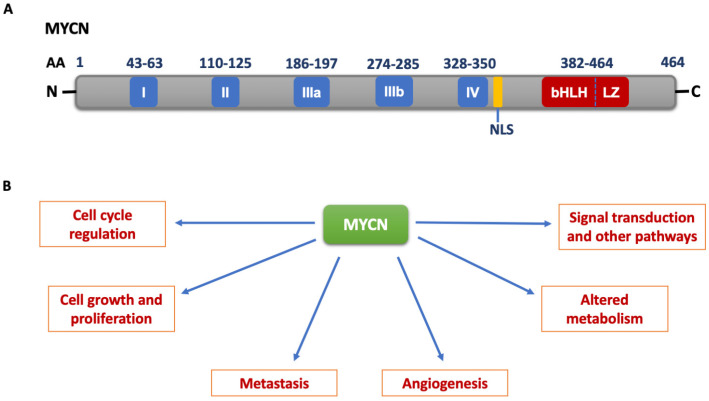
(**A**) MYCN structure. Blue boxes represent the conserved MYC homology boxes; yellow box represents nuclear localization signal (NLS); red box represents the basic Helix-Loop-Helix-Leucine Zipper (bHLH-LZ) domain. AA: amino acid. (**B**) Functions of MYCN in cancers [16,35,36].

The MYCN oncogene, as well as the other MYC family members c-MYC and MYCL, encode nuclear proteins of similar size. The expression and regulation greatly differ between c-MYC and MYCN. c-MYC is expressed ubiquitously in rapidly proliferating cells throughout the developmental process and in adult tissues, whereas MYCN expression is tissue specific. MYCN is specifically expressed in pre-B cells, neuronal tissues, and cells in the intestine, heart, and kidney during embryogenesis [8,17]. MYC proteins contain a C-terminal domain which comprises a DNA binding domain and a basic helix-loop-helix (bHLH) leucine zipper (LZ) domain, which is important for protein-protein interactions and dimerization with other bHLH-LZ proteins such as MYC-associated factor-X (MAX) [18,19,20,21]. The MYC-MAX heterodimer binds to the target DNA in a sequence-specific manner [22]. The N-terminal transcriptional regulatory domain contains a transactivation domain (TAD) in which conserved MYC boxes I and II are present. The central domain consists of three MYC boxes, IIIa, IIIb, and IV, and a nuclear localization sequence (NLS) (Figure 1A) [23,24,25]. The MYC boxes III and IV have functions in regulating the activity of MYC, transformation, and apoptosis [26,27]. The sequence conservation is highest among these five MYC boxes among different MYC family members across different species [28,29,30]. MYC regulates the expression of several genes through binding to the CAC(G/A)TG enhancer box sequences (E-boxes) [31,32]. MYC also binds to the E-box sequences present in the CpG islands, which are also the transcriptional sites [33]. MYCN has several critical functions encompassing cell cycle regulation, cell growth and proliferation, tumor cell angiogenesis, and metastasis. MYCN has also been shown to regulate the expression of various signaling pathways and metabolic enzymes (Figure 1B) [8,34].

## 3. Deregulation of MYCN in Embryonal Neoplasms

Neuroblastoma (NB), retinoblastoma (RB), medulloblastoma (MB), Wilms tumor (WT), and rhabdomyosarcoma (RMS) are examples of embryonal tumors with MYCN dysregulation [16]. These tumors have changes in other molecular pathways in addition to the deregulation of the MYCN oncogene. Neuroblastoma constitutes approximately 8% of all cancers in children and out of which, around 25% are high-risk NBs with MYCN amplification [8]. Retinoblastoma makes up for 4% of childhood tumors [37] and is characterized by MYCN amplification and/or overexpression [38,39,40]. Wilms tumor contributes around 6% to overall childhood neoplasms and shows mutations in MYCN along with copy number gains and overexpression. Both copy number gains and amplification are noted in rhabdomyosarcoma; whereas, MYCN amplification is seen in medulloblastomas. [16,41].

The best possible strategy for limiting tumor cell growth caused by MYCN alterations would be to target MYCN directly. MYCN, on the other hand, lacks the distinct enzymatic activity and pocket for the small-molecule binding that most pharmacological strategies require. Furthermore, as a transcription factor, MYCN is mostly located in the nucleus, making antibody-based therapies ineffective. As a result, MYCN is widely regarded as undruggable. Therefore, it is imperative to drive the efforts to target genes/pathways downstream of MYCN. One such pathway is metabolic reprogramming. Efforts have been intensified in recent times to target alterations in metabolic pathways to constrain tumor cell proliferation.

## 4. Metabolic Reprogramming in Cancer and MYCN

Cancer cells undergo a transformation and display uncontrolled cell growth and proliferation because of changes in various cellular parameters present in normal cells. Transformed tumor cells exhibit several unique characteristics defined as ‘hallmarks’ by Hanahan and Weinberg such as genomic instability, avoiding immune surveillance, opposing cell death, and deregulated cellular metabolic reprogramming among others [42,43].

The MYCN oncogene, similar to its paralog c-MYC, regulates multiple metabolic pathways in RB, NB, and other tumors [40,44,45]. The role of metabolic reprogramming in NB is extensively studied compared to other tumors with MYCN amplification [46]. Here we focus on lipid metabolism and its role in tumor pathogenesis and progression of embryonal tumors with alterations in MYCN comprising NB, RB, MB, RMS, and WT. Many of these embryonal tumors are considered ‘cold tumors’ making them difficult to treat. Direct targeting of MYCN has not been successful due to its intrinsic undruggable nature [47,48,49,50]. As a result, MYCN was targeted indirectly using BET bromodomain inhibitors [51]; however, these compounds can inhibit all the MYC members [52,53]. MYCN was additionally targeted using aurora kinase A inhibitors [54]. Many of these approaches targeting MYC/MYCN have not been successful in clinical trials due to lack of activity or less response rate in vivo, development of drug resistance, dose-limiting toxicity, and/or reprogramming of growth-promoting signaling pathways [55,56,57,58,59,60]. As an alternative approach, molecular signaling pathways downstream of MYCN such as metabolic reprogramming shall be considered.

## 5. Lipid Metabolism

**Figure 2:**
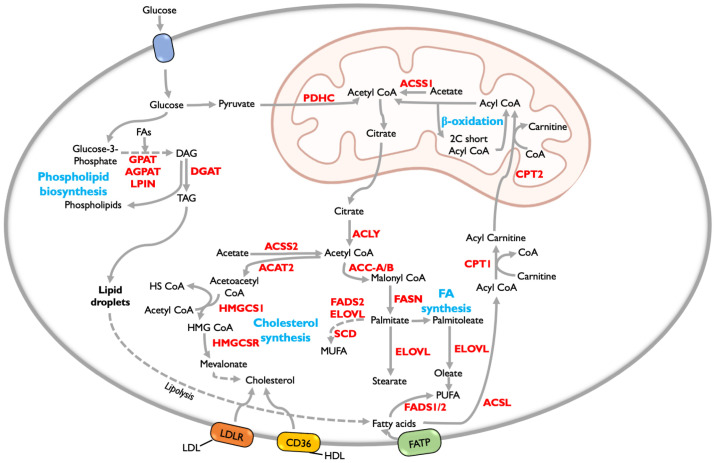
The fundamental biosynthetic pathways of lipid metabolism in cancer [46,77,78]. The red color indicates the important enzymes involved in lipid metabolism. The blue color indicates the key lipid metabolic pathways. ACSS1/2—acyl-coenzyme A synthetase short-chain family member 1/2; ACSL—long chain acyl CoA synthase; ACAT2—acetyl CoA acetyl transferase 2; AGPAT2—1-acylglycerol-3-phosphate O-acyl transferase 2; ACC-A/B—acetyl CoA carboxylase A/B; CPT1/2—carnitine palmitoyl transferase 1/2; CD36—cluster of differentiation 36; DGAT—diglyceride acyltransferase; ELOVL—elongation of very long chain fatty acids; FASN—fatty acid synthase complex; FADS1/2—fatty acid desaturase 1/2; FATP—fatty acid transport protein; GPAT—glycerol-3-phosphate acyl transferase; HMGCR—3-hydroxy-3-methylglutaryl CoA reductase; HMGCS1—3-hydroxy-3-methylglutaryl CoA synthase 1; LPIN—lipin; LDL—low-density lipoprotein; LDLR—low-density lipoprotein receptor; PDHC—pyruvate dehydrogenase complex; SCD—stearoyl-CoA desaturase. A few of the icons were used from Biorender.com (accessed on 16 March 2023).

Lipids are one of the major classes of biomolecules required for cell growth and survival. Lipids fulfill the basic requirement of a cell such as energy production, signal transduction, and membrane synthesis that aid in the proliferation and metastasis of cancer cells [61,62]. Phospholipids, glycerolipids, sphingolipids, glycolipids, sterols, fatty acids (FA), and their derivatives are some examples of lipid molecules. Sphingolipids, diacylglycerides, and triglycerides act as signaling and storage molecules, while phospholipids and cholesterol make up the majority of the plasma membrane [63]. Cancer cells obtain the majority of the lipids by de novo lipogenesis, whereas normal cells utilize exogenous lipids [64]. Tumors with MYC or MYCN alterations have higher levels of lipids and their levels correlate with poor patient outcomes [65]. Cancer cells employ all major lipid metabolic pathways including biogenesis, uptake, oxidation, storage in the form of lipid droplets, and membrane biosynthesis (Figure 2) [66]. Lipid metabolic reactions in tumor cells obtain ATP required for their biosynthesis from aerobic glycolysis/Warburg effect [67]. Altered lipid metabolism also plays a significant role in cancer hallmarks such as enhanced cell proliferation, angiogenesis, and escaping immune response among others [62].

Interestingly, cancer stem cells (CSCs) may also share similar lipid metabolic reactions [68], but their regulation may differ. For instance, Nanog, a regulator of stem cell self-renewal supports beta-oxidation in tumor cells [69]. In hepatocellular carcinoma, Nanog activates fatty acid oxidation [69]. Similarly, stearoyl Coenzyme A (CoA) desaturase (SCD1) that catalyzes the desaturation of fatty acids has been shown to be important for CSC maintenance [70,71]. Deregulation of lipid metabolism is also observed in oral CSCs that shows increased expression of CD36, a fatty acid transporter [72]. Further, a high amount of lipid droplets was found in colorectal CSCs [73]. Two key enzymes of lipid metabolism, SCD1, and 3-hydroxy-3-methylglutaryl-CoA reductase (HMGCR), a key enzyme in cholesterol biosynthesis were found to be associated with the fate of CSCs. Both the enzymes activate Yes1 associated protein (YAP)/phospholipid-lysophospholipid transacylase, TAZ oncogene pathway in CSCs [74,75]. In lung CSCs, SCD1 inhibition regresses chemoresistance [76]. Overall targeting lipid metabolism in CSCs would help to eliminate them in various tumors.

### 5.1. Fatty Acid Metabolism

Acetyl CoA is the starting molecule for fatty acid synthesis, with glutamine and glucose serving as the primary sources. Glucose enters the cell via glucose transporters and is converted to pyruvate by the glycolytic pathway. Pyruvate enters mitochondria where it is catalyzed by pyruvate dehydrogenase complex (PDHC) to form acetyl CoA that enters the tricarboxylic acid (TCA) cycle and forms citrate [79]. Citrate is transported out to cytosol, where it is catalyzed by ATP citrate lyase (ACLY) to form cytosolic acetyl CoA. ACLY has been found to be overexpressed in breast cancer, lung cancer, hepatocellular carcinoma, colon cancer, and rectal cancer [80,81,82,83]. Alternatively, acetyl CoA can be directly formed from acetate mediated by acyl-coenzyme A synthetase short-chain family member 2 (ACSS2) [84]. Reductive carboxylation of acetyl CoA by acetyl CoA carboxylases (ACC-A/B) results in the formation of malonyl CoA. ACC is the rate-limiting enzyme in the synthesis of fatty acids in tumors and regulates metabolism in pancreatic cancer, non-small cell lung cancer, and liver cancer [85,86,87]. The fatty acid synthase (FASN) complex catalyzes the formation of palmitate, the most abundant saturated fatty acid, from malonyl CoA (Figure 2). FASN has been identified as an oncogene in gliomas and cancers of the breast, colon, prostate, lung, skin, and bladder [88,89]. Suppressing FASN expression may lead to cell death as studied in ovarian cancer [90].

Fatty acid desaturases (FADS), SCD, and elongation of very long chain fatty acid (ELOVL) proteins act on palmitate to produce monounsaturated (MUFA), polyunsaturated (PUFA), and very long chain fatty acids that serve as a storage pool of fatty acids (Figure 2) [91,92]. The balance of FA saturation and desaturation is crucial for cell survival in cancer since enhanced levels of saturated fatty acids lead to toxicity [93]. So, they are either converted to lipid droplets for storage or phospholipids that are incorporated into the membrane [93]. Furthermore, elevated unsaturated fatty acids in the membrane cause ferroptosis due to oxidative deterioration of lipids [94,95,96]. SCD was found to be overexpressed in multiple cancers and corresponds to decreased cell survival [97]. SCD requires oxygen to catalyze the desaturation reaction, so less unsaturated FAs are found in hypoxic conditions, resulting in decreased tumor cell viability [98]. Reduced expression of SCD or its inhibition induces endoplasmic reticulum (ER) stress and cell death in glioblastoma [99]. Similarly, SCD1 inhibition in lung and colon cancers results in cell death. In addition to SCD1, FADS2 is involved in the desaturation of essential fatty acids such as linoleic acid to produce PUFA and the conversion of palmitate to sapienate [100]. Interestingly, when SCD1 was inhibited in vitro in liver and lung cancer cells, there was no cell death until FADS2 was silenced. FADS2 acts as a substitute to carry out desaturation reactions in these tumors that are resistant to SCD inhibition [100]. Further, levels of MUFA and PUFA are highly altered in cancers such as hepatocellular carcinoma, urothelial carcinoma, lymphoma, and colorectal and prostate cancers [101,102,103,104,105]. It was observed that ꞷ3-PUFA induces cell death in breast cancer and glioblastoma cells and may be used as a therapeutic drug for treatment [106,107]. Lipids in low-density lipoproteins (LDL) are also taken up by the cell via LDL receptors and are catalyzed by lipases to form the pool of essential fatty acids [108].

### 5.2. Fatty Acid Oxidation (FAO)

Fatty acids are oxidized in mitochondria when there is an energy requirement in a cell. It is also known as beta-oxidation since the carbon at the beta position of fatty acid is oxidized. Beta-oxidation utilizes acyl-CoA as the starting material. Long chain acyl CoA synthase (ACSL) adds CoA to fatty acids present in cytosol forming acyl CoA. Next, a carnitine group is transferred to acyl CoA by carnitine palmitoyl transferase 1 (CPT1) to form acyl carnitine. Acyl carnitine enters the mitochondria where carnitine palmitoyl transferase 2 (CPT2) removes carnitine from acyl carnitine to form acyl CoA (Figure 2). FAO occurs in four major steps, dehydrogenation, hydration, oxidation, and thiolysis, as a result of which, acyl CoA is broken down to acetyl CoA. In addition, NADH, FADH2, and ATP are also produced. Acetyl CoA can enter the TCA cycle for further energy production [109]. ATP produced by fatty acid oxidation is almost twice that of glucose and hence is a significant energy source for cancer cells [110]. It has been studied in many cancer cells that the oxidation of fatty acids acts as the energy source for survival [111,112]. ACSL protein family consists of five members and they have differential overexpression in various tumors [113]. CPT1A, a largely distributed isoform of the CPT family, is highly expressed in many cancers. CPT1A helps the cancer cells to survive against stresses such as hypoxia, limited nutrition, and radiation [114]. CPT2 has been found to be a biomarker in colon cancer and hepatocellular carcinoma [115,116]. It has been demonstrated that inhibition of fatty acid oxidation causes oxidative stress, which results in cell death and loss of adherence in solid tumors [117,118].

### 5.3. Phospholipid Synthesis

Phospholipids, which include phosphatidyl serine (PS), phosphatidyl choline (PC), phosphatidyl inositol (PI), and phosphatidyl ethanolamine (PE) are the most abundant components of the plasma membrane. Phospholipids are classified into two types: glycerophospholipids with glycerol as the backbone and sphingophospholipids with sphingosine as the backbone [119]. Glycerol-3-phosphate, a by-product of glycolysis, serves as the starting point for the production of phospholipids. Glycerol-3-phosphate acyl transferase (GPAT) adds an acyl group to glycerol-3-phosphate, forming lysophosphatidic acid (LPA). GPAT1 has been found to be a biomarker in ovarian cancer [120]. The enzyme 1-acylglycerol-3-phosphate O-acyl transferase (AGPAT) catalyzes the formation of phosphatidic acid (PA) from LPA. Upregulation of AGPAT has been observed in colon and ovarian cancers [121,122]. Phosphatidic acid phosphatase, lipin (LPIN) acts on PA to form diacylglycerol (Figure 2). It has been uncovered that the expression of LPIN1 correlates with an unfavorable prognosis in triple-negative breast cancer (TNBC) and lung adenocarcinoma [123,124]. Cytidine-5′-diphosphate (CDP) diacylglycerol (DAG) synthase catalyzes the formation of CDP-diacylglycerol from PA and cytidine-5′-triphosphate (CTP) [125]. DAG and CDP-diacylglycerol act as substrates for PC and PE, and PI and PS synthesis respectively [125]. Phospholipids are transported to different organelles after synthesis in the ER by transport proteins such as PC-specific transporter, sterol carrier protein 2, and PI transfer protein [126,127,128]. The activity of the transporters is altered in tumors, thereby changing the composition of phospholipids in the membrane and aiding in tumorigenesis [129,130]. Finally, diglyceride acyltransferase (DGAT) converts diacylglycerides to triacylglycerides (TAGs) [131]. TAGs may be stored as lipid droplets for later use. Lipoprotein lipases (LPL) can hydrolyze TAGs to form FAs for energy production (Figure 2). DAG and fatty acids are also produced via the hydroxylation of TAG by adipose triglyceride lipase (ATGL).

### 5.4. Cholesterol Synthesis

Cholesterol is a crucial lipid for the membrane because it helps to maintain membrane fluidity and the formation of lipid rafts [132]. It can be produced endogenously or absorbed from food [133,134]. Acetate is the starting point of cholesterogenesis that occurs in the cytoplasm. Two acetyl CoAs condense to form acetoacetyl CoA by acetyl-CoA acetyl transferase (ACAT1). ACAT1 is being studied as a biomarker in aggressive breast and prostate tumors [135,136]. The enzyme 3-hydroxy-3-methylglutaryl CoA synthetase (HMGCS) catalyzes the addition of acetyl CoA to acetoacetyl CoA to produce 3-hydroxy-3-methylglutaryl (HMG) CoA. HMGCS2 is thought to be a significant target in advanced cancers [137]. The rate-limiting step of cholesterol synthesis is the mevalonate formation upon reduction of HMG CoA by HMG CoA reductase (HMGCR) (Figure 2). HMGCR has been shown to be a good prognostic marker in colon and breast cancers, and it correlates with a better clinical outcome [138,139].

## 6. Lipid Metabolism in Embryonal Tumors

Traditionally, lipid metabolism was studied in relation to metabolic diseases such as obesity and hyperlipidemia. However, the recent literature suggests a role for lipid metabolism in tumorigenesis and evidence further supports that the deregulation of lipid metabolism modulates downstream signaling pathways and oxidative stress in cancer [140]. In the next few sections, we discuss the various lipid metabolic reactions that were found to be altered in individual embryonal tumors with MYCN dysregulation.

### 6.1. Neuroblastoma

Neuroblastoma is an extra-cranial tumor of the sympathetic nervous system arising in the precursor cells of the neural crest region that contributes to 13–15% of all cancer-related deaths in children [141,142]. Neuroblastoma has different molecular subgroups based on the nature of genetic alterations. Amplification of MYCN oncogene has been found in a subset of high-risk NB and these patients have advanced disease coupled with poor prognosis [12,13,143]. Anaplastic lymphoma kinase (ALK) [144] is among other genes that contribute to NB pathogenesis.

The de novo lipogenic FASN enzyme is shown to be expressed at a higher level in the NB cell line SK-N-SH compared to the Hs27 fibroblast cell line [145]. Though the expression of FASN is not thoroughly studied in NB patient specimens, the functional significance of FASN is studied using small molecule inhibitors that suggests a critical role of FASN in NB tumorigenesis. Similarly, the importance of the ACC-A enzyme has also been studied using pharmacological inhibition [146]. Different cell models with altered MYCN levels also specifically show that glycerolipids particularly DAG is the most abundant lipid in MYCN amplified NB [45], followed by phospholipids. The accumulation of DAG appears to be an important protective mechanism for the tumor cells as it incorporates free fatty acids into TAG and lipid droplets and this phenomenon is mediated by MYCN via the upregulation of DGAT [45]. Interestingly, MYCN also represses the accumulation of anti-tumorigenic long-chain fatty acid docosahexanoic acid via repressing the expression of fatty acid elongase, ELOVL2 [147]. On the contrary, it has been shown that MYCN inhibition resulted in the accumulation of lipid droplets in NB cells as a result of inhibition of beta-oxidation [148]. Furthermore, an essential enzyme in fatty acid beta-oxidation, CPT1C is overexpressed in NB, and that correlated with poor prognosis. Pharmacological inhibition of CPT1C provided evidence that it could be a potential drug target [149]. Additionally, gene expression data from transgenic mouse models that drive the expression of MYCN under tyrosine hydroxylase gene promoter showed that the genes involved in cholesterol biosynthetic mevalonate pathway, HMGCS1, HMGCR, and mevalonate kinase (MVK) were elevated by the concerted action of MYCN and sterol regulatory-element binding factor (SREBF) [150].

**Figure 3:**
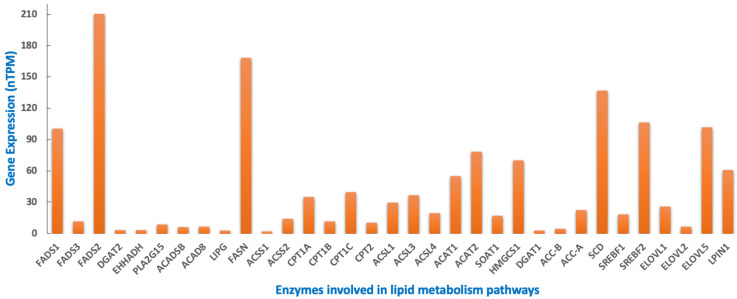
Gene expression profile of lipid metabolic enzymes in Neuroblastoma. The median expression of various lipid metabolic enzymes found in neuroblastoma was retrieved from Human Protein Atlas [150,151]. nTPM—number of transcripts per million; nTPM > 1 is considered to be a cancer-specific enhanced expression in multiple cell lines. ACSS1/2—acyl CoA synthetase short-chain family member 1/2; ACSL—long chain acyl CoA synthases; ACAT2—acetyl CoA acetyl transferase 2; ACC-A/B—acetyl CoA carboxylase A/B; CPT1/2—carnitine palmitoyl transferase 1/2; DGAT—diglyceride acyltransferase; ELOVL—elongation of very long chain fatty acids; FASN—fatty acid synthase complex; FADS—fatty acid desaturase; HMGCS—3-hydroxy-3-methylglutaryl CoA synthase; LPIN—lipin; SCD—stearoyl CoA desaturase; SREBF—sterol regulatory element binding factor; EHHADH—enoyl-CoA hydratase and 3-hydroxyacyl CoA dehydrogenase; PLA2G15—phospholipase A2 Group XV; ACAD—acyl-CoA dehydrogenase short-chain; LIPG—lipase G; SOAT1—sterol O-acyltransferase 1.

Further, to identify the different dysregulated genes in lipid metabolism in NB, we have analyzed the expression data retrieved from the human protein atlas [150,151]. The data show that fatty acid synthase, FASN, and desaturases, FADS1 and FADS2 are the most highly expressed genes in NB. Other enzymes with moderately high expression include carnitine palmitoyl transferases, CPT1A and CPT1C, acetyl CoA acetyl transferases, ACAT1 and ACAT2 and HMG-CoA synthase, HMGCS1 (Figure 3). The above findings require further validation in NB patient specimens and the potential of targeting them needs to be explored in the future.

In addition to de novo fatty acid synthesis, fatty acid uptake provides fatty acids required for biosynthetic processes. Fatty acids are taken up by the membrane-bound CD36 fatty acid translocase and fatty acid transport proteins, FATP1–6, encoded by SLC27A1–6 genes and distributed within the cells by fatty acid binding proteins (FABP1–12) [152]. NB cells with MYCN amplification seem to depend on both import of long-chain fatty acids via fatty acid transport protein 2 (FATP2) and fatty acid synthesis for their survival. Interestingly, targeting fatty acid uptake sensitized NB cells to routine chemotherapy drugs, which, shows that FA uptake is necessary for NB cell survival [45]. Overall, the existing literature suggests that targeting lipid metabolism in NB can be exploited further to develop novel treatment strategies.

### 6.2. Retinoblastoma

A pediatric neoplasm originating in the retina of infants and children. Retinoblastoma is usually initiated by the loss of function of the RB1 tumor suppressor gene [153,154]. Recent studies show that MYCN amplification and/or overexpression is an immediate event following RB1 inactivation [155,156]. Additionally, there is a subgroup of RB that exhibits MYCN amplification without the RB1 gene mutations [39]. Earlier studies have shown that glucose metabolism is highly regulated by MYCN in retinoblastoma [40]. However, the knowledge of lipid metabolism is rather limited in RB. Recent metabolite analysis identified differential metabolites in RB and the total lipid content is elevated in a subgroup of patients that correlated with the extent of necrosis in tumor samples [157]. Systems-level metabolite analysis further predicts lower utilization of cholesterol; possibly to make NADPH available for other biochemical reactions [158]. At the individual metabolic reactions level, the expression and possibility of therapeutic targeting of FASN were extensively studied in RB. The expression of FASN was found in 82% of patient samples that were shown to be associated with advanced disease features such as optic nerve and choroid invasion, poor differentiation, and high mitotic index [159]. Further, lipid composition was found to be heterogenous between two different cell lines of RB indicating the possibility of a different cell of origin [160]. Genetic knockdown of FASN in RB further confirmed its role in tumor cell proliferation and progression [161]. Inhibition of FASN in RB cell line models resulted in enhanced apoptosis suggesting that FASN could be a therapeutic target in RB [162]. Future studies are required to profoundly understand the role of various other lipid metabolic reactions in RB, particularly, their regulation by the MYCN oncogene.

### 6.3. Medulloblastoma

Medulloblastoma (MB) is an aggressive brain cancer originating in the cerebellum of children. Medulloblastoma has four subtypes, WNT (wingless), Shh (sonic hedgehog), group 3, and group 4 [163,164,165]. Different subgroups have alterations in different members of the MYC family. Though lipid metabolism is not specifically studied in MYC-dependent MB, all the subtypes of MB may likely regulate metabolic events through convergent action of MYCN or MYC. Lipid metabolism was however studied in a transgenic mouse model of MB that harbors a mutant smoothened allele in which the sonic hedgehog pathway was dysregulated [166]. Around two-thirds of mice developed MB. The tumor area exhibited elevated expression of FASN and increased de novo fatty acid biosynthesis coupled with decreased fatty acid oxidation. The elevated expression of FASN also correlated with the expression of transcription factors E2F1 and MYCN. Interestingly, these mice with MB when treated with fatty acid synthesis inhibitor C75 showed increased survival and decreased lipid synthesis along with decreased expression of proliferation markers MYCN and cyclin D2. Acetyl CoA carboxylase was also found to be dysregulated in this mouse model [166]. On the contrary, lipid catabolic enzymes were found to be downregulated in this model [166]. Further, it was also observed that Shh-dependent MB mouse models show differences in lipid composition between primary and metastatic samples, possibly reflecting the differences in the requirement for metastasizing cells from primary tumor cells [167].

The gene expression data in MB patient samples from Medulloblastoma Advanced Genomic International Consortium (MAGIC) database showed that Shh, Group 3, and Group 4 MBs have elevated expression of FASN and SCD [168]. On the other hand, a different study has shown that lipid levels are less in Group 3/4 MBs compared to the Shh group [169]. However, comparison with control tissues may still reveal elevated levels of lipids in these MB subgroups as shown before. MBs also show elevated cholesterol biosynthesis and thus raising the possibility of using statins in the treatment of MBs [170]. Interestingly, it has been noticed that cerebrospinal fluid (CSF) from MB patients had higher levels of triglycerides compared to CSF from children with no cancer [171]. Tissue analysis of metabolites showed that MBs have higher lipid levels compared to other atypical teratoid/rhabdoid tumors [172]. It has been shown that MYCN could be a downstream target of the shh pathway in MB and its role in the regulation of glycolytic metabolism was studied in MB models [166,173]. However, the role of MYCN in the regulation of lipid metabolism still needs to be explored. Interestingly, these lipid metabolic events were shared between cerebellar granule neural progenitors and MB cells [174,175]. Additionally, MB cells that show acquired resistance to BET bromodomain inhibitors have elevated levels of lipid metabolic enzymes; suggesting that targeting lipid metabolism might be a good strategy in these resistant tumor cells [176].

### 6.4. Rhabdomyosarcoma

Rhabdomyosarcoma (RMS) is the most common soft tissue neoplasm in children and adolescents. Based on histology, alveolar and embryonal subtypes were described. The predominant subtypes harbor Pax3/7-Foxo1 fusion transcript (commonly referred to as fusion-positive) or show mutation/s in RAS/PI3K/AKT pathways (usually called fusion negative) [177,178,179,180]. It has been observed that Pax3-Foxo1 drives the expression of the MYCN oncogene [181]. It is also known that the Pax3-Foxo1 fusion protein can activate the expression of a rate-limiting enzyme in fatty acid beta-oxidation, CPT1A by directly binding to its promoter region [182]. However, the expression of fatty acid biosynthetic enzymes and their role in RMS pathogenesis is not completely understood. Nevertheless, RMS cell lines when treated with an inhibitor of malonyl-CoA decarboxylase (CBM-3001106) that enhances malonyl-CoA levels and decreases fatty acid beta-oxidation showed reduced cell proliferation by forcing the cells to enter into cell cycle arrest [183]. Further studies are required to completely decipher the functional significance of altered lipid metabolism in RMS. Particularly, studies on primary patient samples are greatly required.

### 6.5. Wilms Tumor

A typical childhood malignant tumor arising in the kidneys accounts for 6% of all childhood cancers [184]. Wilms tumor (WT), also denoted as nephroblastoma is mostly unilateral in nature with 5–6% of cases being bilateral [185]. In addition to WT1, FBXW7, and TP53 gene mutations, a gain of MYCN copies was found in 8.7% of cases that are frequently found in the high-risk subgroups with diffuse anaplasia [186,187]. In addition to copy number gain, MYCN was also found to contain activating point mutation in Wilms tumor. MYCN copy number gain is also associated with poor overall survival [188]. MYCN was also found to be overexpressed in Wilms tumor [188].

Proteomic analysis of tissues from WT and the adjacent healthy area identified several differentially expressed enzymes involved in lipid metabolism. Among these, three proteins, FASN, inositol-3-phosphate synthase 1 (ISYNA1), and arachidonate-15-lipoxygenase (ALOX15) were found to be considerably upregulated. Notably, further analysis involving immunohistochemistry and Kaplan-Meier analysis identified a poor prognostic value for FASN expression. The expression of FASN also correlated with tumor stage and size indicating its prognostic value as a biomarker for tumor progression [189]. On the contrary, proteins involved in long-chain fatty acid metabolism were found to be reduced in WT samples that were collected from chemotherapy-treated patients [190]. Further, induction or suppression of WT1 in cell line models identified genes regulated by WT1 tumor suppressor. This data identified the regulation of genes associated with fatty acid and cholesterol metabolism. In addition, it was speculated that WT1 regulates sterol regulatory element binding proteins (SREBPs) to alter mevalonate pathway genes involved in the production of cholesterol and cholesterol-related lipids [191]. Overall, a lack of studies suggests that further analyses are needed to understand lipid metabolism in Wilms tumor and specifically in different subtypes resulting from different gene alterations.

## 7. Targeting Lipid Metabolism as a Therapeutic Strategy

Lipid metabolic reprogramming is currently being investigated for specific targeting of tumor cells. The overexpression of several crucial enzymes involved in lipid metabolism lends support for the development of small molecules to specifically inhibit them. In this section, we discuss various small molecule inhibitors that can target lipid metabolism in cancer.

The key enzymes of lipid biosynthesis, FASN, ACC, and SCD1 play important roles in the tumorigenesis of various cancers. Blocking FASN will reduce the fatty acid synthesis and thus fatty acid oxidation. Some of the FASN inhibitors include TVB-2640, TVB-3166, TVB-3664, C75, cerulenin, and orlistat. TVB-2640, TVB-3166, and TVB-3664 are orally available reversible FASN inhibitors. TVB-2640 is in phase II clinical study in non-small cell lung carcinoma with KRAS mutation (NCT03808558) [192,193]. It is evident that TVB-3166 mediates cell death by altering PI3K/AKT/mTOR, ꞵ-catenin, and c-MYC signaling pathways [77,194]. On the other hand, orlistat binds irreversibly to the thioesterase domain of FASN and inhibits the xenograft growth of prostate cancer cells [195]. Although orlistat is an approved anti-obesity drug (NCT05496075), there are no ongoing clinical trials in cancers using this drug [77].

ACC is another rate-limiting enzyme in fatty acid synthesis. Allosteric inhibitors of ACC consisting of ND-646, ND-654, 5-tetradecyloxy-2-furoic acid (TOFA), and soraphen A are in preclinical trials. ND-646 was found to be an allosteric inhibitor of ACC in non-small cell lung cancer [86]. TOFA is also an allosteric inhibitor of ACC that induces apoptosis in multiple cancer cells [196]. In a murine model of HCC, ND-654 was found to reduce tumor development by mimicking ACC phosphorylation and thereby hindering lipid synthesis [87,197]. Phosphorylation of ACC typically inhibits its enzymatic activity resulting in decreased lipid synthesis.

SCD1 inhibitors include A939572 and CAY10566; A939572 induces ER stress in renal cell carcinoma [198] and CAY10566 treatment leads to apoptosis in hepatocellular carcinoma [199]. A939572 is a nicotinamide derivative that inhibits colon cancer and ovarian cancer xenograft tissue growth [77,200,201]; it also reduces PI3K/AKT phosphorylation leading to cell death in lung cancer cells [202]. CAY10566, available orally, has shown promising anticancer effects in glioblastoma xenografts [203] and prevents ovarian cancer spheroid formation by reducing PI3K/AKT phosphorylation [70]. BZ36, another nicotinamide-derived inhibitor of SCD1 has demonstrated anti-tumor activity against prostate cancer cells and xenografts [204]. Compound-3j and MK-8245 that block SCD1 based on its structural activity are specific to the liver lipid profile [99,205]. MK-8245 is also shown to have anti-proliferative properties in a zebrafish model of liver cancer [205].

Drugs used against other lipid metabolic enzymes such as HMGCR include statins that have been shown to be potential anticancer agents [206]. Statins are found to inhibit the mevalonate pathway, induce oxidative stress, and increase apoptosis in multiple cancers [207]. In combination with chemotherapeutics, statins have shown encouraging results in clinical trials [208,209,210,211]. Interestingly, statins when combined with other anti-cancer molecular therapeutics prevail over drug resistance mechanisms [207]. Etomoxir is an irreversible inhibitor of CPT1, shown to inhibit high MYC-expressing triple negative breast cancer cells [112]. Perhexiline is a competitive CPT1 inhibitor, that blocks cell growth in breast cancer and glioblastoma [212,213].

Additionally, compounds targeting SREBPs include silibinin which promotes autophagy in breast cancer cells [214]; fatostatin hinders cell growth in breast cancer, and glioblastoma [215,216]; an improved compound of fatostatin is compound 24 that inhibits SREBP activity resulting in reduced cholesterol synthesis in glioma cells [217]; and nelfinavir hinders cell growth in prostate cancer [218]. The few drugs mentioned above are yet to be explored to inhibit lipogenesis in childhood cancers with MYCN dysregulation.

Chemotherapy drugs used against cancer often leave side effects in the patient due to toxicity towards normal, healthy, and unaffected cells. So, a combination of drugs with small molecule inhibitors is found to be beneficial in multiple cancers, since the drug concentration gets reduced. For instance, a combination of FASN inhibitors with paclitaxel and docetaxel in lung and colon cancer cells improved antitumor effects in comparison to the use of only a single drug [219].

Compounds derived from natural products will have the least toxicity. Few such compounds are already being studied and explored for anticancer properties. Examples include berberine, a plant-based alkaloid that decreased FASN expression in breast cancer cells [220]; betulinic acid obtained from barks of various plants, works by inhibiting the activity of SCD1 in cervical cancer cells [221], and curcumin reduced lipid droplets formation in glioblastoma cells [222].

## 8. MYCN Regulation of Lipid Metabolism

The role of c-MYC in metabolic reprogramming has been extensively studied [7,223]. MYCN, similarly, has been shown to have a regulatory role in various metabolic pathways. For example, MYCN has been shown to have a role in the glycolytic pathway in RB [40,224]. Likewise, MYCN plays a similar role in NB with a regulatory role in the glycolytic pathway, glucose oxidation, and glutamine metabolism [149]. Similarly, MYCN upregulates the expression of other metabolic proteins such as amino acid transporter ASCT2 [225]. In addition to metabolic pathways, MYCN is shown to regulate multiple other pathways such as cell cycle regulation [226,227]. The role of MYCN in the regulation of lipid metabolism is currently an active area of investigation.

The lipid biosynthetic pathway is typically regulated by SREBPs. They are transcription factors that regulate the genes associated with lipid metabolism [228]. There are two isoforms found; SREBP1 is involved with FA metabolic genes, whereas, SREBP2 controls cholesterol synthesis genes [229,230]. SREBP1 has recently been identified as a potential therapeutic target in prostate cancer, breast cancer, and glioblastoma [231,232,233]. Important signaling pathways such as EGFR, PI3K/AKT/mTOR, and others activate SREBPs by inducing proteolytic cleavage of its N-terminal domain, thus promoting tumorigenesis [232,234]. SREBP2 turns on the mevalonate pathway by activating key enzymes such as HMGCR in tumors including NB [235,236]. Mevalonate subsequently activates YAP/TAZ pathway and stimulates cell proliferation and survival in tumors. YAP and TAZ are important transcription coactivators that regulate the hippo signaling and are crucial for cell growth and proliferation [75]. It was found that SREBP is important for the regulation of lipogenesis by MYC in tumors such as renal carcinoma, hepatocellular carcinoma, and lung cancer [237,238,239]. SREBP2 in cancer cells is localized to the nucleus due to low extracellular pH, where it targets ACSS2 for cell survival [240]. The expression of SREBPs may be regulated by MYCN as well. However, another transcription factor that belongs to the MYC superfamily, MondoA, in addition, either independently regulates the expression of SREBPs, acetyl CoA synthases ACSL3, and ACSL4, fatty acid elongases, ELOVL5, and ELOVL6, SCD, FASN, and diacylglycerol acyltransferases, DGAT1, and DGAT2 [241], or cooperates with MYCN in the regulation of few other biosynthetic pathways [241] possibly by binding to E-box sites present on the promoter region of these genes. MYCN regulates the expression of MondoA and the expression of MondoA is required to maintain the levels of SREBPs to regulate lipid biosynthesis [241]. The function of MondoA in NB is established, but there could be a role for MondoA in other MYCN-amplified childhood cancers as well. MYCN amplified NBs also show decreased production of anti-tumorigenic ꞷ-3 fatty acid docosahexanoic acid (DHA). MYCN has been shown to block the activity of ELOVL2, a key enzyme in DHA synthesis along with the recruitment of PRC1 (Polycomb repressive complex 1) [147].

**Figure 4:**
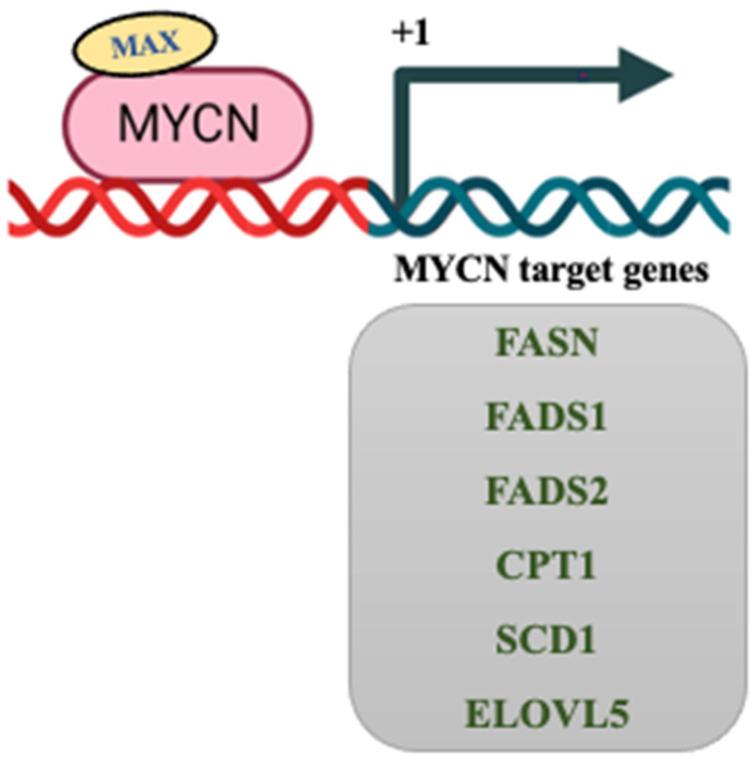
Possible regulation of key lipid metabolic enzymes by MYCN. The promoter sequence of important lipid metabolic enzymes was analyzed for the presence of MYC-binding E-boxes. The few key enzymes possibly regulated by MYCN are shown. MYCN—MYC- neuroblastoma derived; MAX—MYC associated factor X; FASN—fatty acid synthase; FADS1/2—fatty acid desaturase 1/2; CPT1—carnitine palmitoyl transferase 1; SCD1—stearoyl-CoA desaturase 1; ELOVL5—elongation of very long chain fatty acids 5. DNA double-strand icon was taken from Biorender.com (accessed on 16 March 2023).

**Figure 5:**
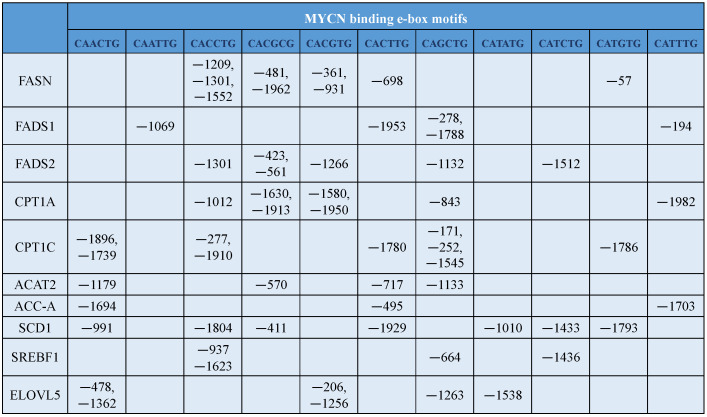
The location of MYCN binding E-box motifs on the promoter region of lipid metabolic enzymes. FASN—fatty acid synthase; FADS1—fatty acid desaturase 1; CPT1—carnitine palmitoyl transferase 1; ACAT2—acetyl CoA acetyltransferase; ACC-A—acetyl CoA carboxylase—A; SCD1—stearoyl-CoA desaturase 1; SREBF—sterol regulatory element binding factor; ELOVL5—elongation of very long chain fatty acids 5.

Further to understand the regulation of lipid metabolism by MYCN, we analyzed the promoter sequences of important rate-limiting lipid metabolic enzymes for the presence of presumed MYC binding sites using the eukaryotic promoter database (EPD) [242]. The analysis shows that FASN, FADS1, FADS2, CPT1C, ACAT2, and ACC-A have MYC-binding E-boxes (Figure 4). The predicted positions of MYCN binding on gene promoters of these enzymes are mentioned in (Figure 5). Overall, this analysis shows that MYCN can directly bind to the E-boxes present on the promoter sequences of the lipid enzymes to regulate their expression. Some of the enzymes and their regulation by MYC/MYCN have been verified [45,243]. For instance, chromatin immunoprecipitation (ChIP) data has shown that MYC binds to the promoter regions of key lipid metabolic enzymes, ACLY, ACC, and FASN in prostate cancer [243]. Similarly, in multiple myeloma, MYC has been shown to bind at the promoters of ACC to regulate its expression [244]. Further, MYCN has been shown to repress the expression of ELOVL2 [147] and ELOVL4 by directly binding at their promoter region. Enhanced expression of ELOVL4 has been associated with good prognosis in NB [245]. Future studies are required to validate the MYC/MYCN mediated regulation of other lipid metabolic enzymes.

On the contrary, the levels of MYCN were in turn shown to be regulated by fatty acid desaturation. For example, when hepatocellular carcinoma cells were treated with small molecule or shRNA against SCD1 led to decreased expression of MYCN and reduced cell proliferation. This suggests the regulation of MYCN expression by lipid desaturation [246]. In addition, enhanced accumulation of inflammation-causing lipids was shown to upregulate the expression of MYCN [247].

Additionally, the role of non-coding RNAs, particularly the role of miRNAs in the regulation of lipid metabolism has been explored. miR-122 was the first identified miRNA to be involved in lipid metabolism. Reduced miR-122 expression led to reduced HMGCS1 and HMGCR expression. miR-122 downregulation is also related to HCC [248]. miR-33a and miR-33b are intronic-derived miRNAs formed from SREBP2 and SREBP1 expressing genes, respectively and thus linking miRNA regulation to lipid metabolism. Intriguingly, SREBPs and CPT1A are the target genes of these miRNAs [249,250,251]. miR-27a represses FASN, SREBP1, and SREBP2 in human hepatoma cells [252]. Further, miR-370 regulates the expression of miR-122 as well as CPT1A in hepatoma cells [253]. Few long non-coding RNAs are also known to target lipid enzymes. For example, lncRNAPCA3 disrupts lipid metabolism in prostate cancer through the miR-132-3p/SREBP1 pathway [254]. lncRNASNHG7 acts as an oncogene in thyroid cancer and needs ACSL1 for tumor advancement [255]. Though non-coding RNA-mediated regulation of lipid metabolism is studied, it is yet to be investigated in pediatric cancers.

## 9. Conclusions

Embryonal tumors with MYCN deregulation such as neuroblastoma, retinoblastoma, medulloblastoma, Wilms tumor, and rhabdomyosarcoma are highly malignant and are often less amenable to targeted therapies. Metabolic reprogramming, being one of the tumor cell hallmarks might possibly be exploited for developing novel drug targets. Lipid metabolism provides energy, membrane components, and signaling molecules for rapidly proliferating tumor cells. The regulation of lipid metabolism is usually mediated by SREBPs; whose expression in turn is regulated by various well-established oncogenic pathways including MYC, EGFR, and PI3K/AKT. However, there are different molecular subtypes in these embryonal tumors. We need to study lipid metabolic alterations in the individual tumor subtypes as well as develop efficacious small molecule inhibitors with fewer side effects and toxicity to specifically target lipid metabolism. In addition, the role of lipid metabolism in developing drug resistance and cancer stem cells needs to be considered in these tumors. Combination therapies involving inhibitors of fatty acid synthesis, desaturation, and/or uptake with chemotherapy drugs would be worthwhile to be tested. Studying lipid metabolism might provide unique opportunities for targeting embryonal tumors with dysregulated MYCN oncogene.
